# Supplementary material for: The Relationship between Oxidative Stress and Subjective Sleep Quality in People with Coronary Artery Disease
Source: Brain Sci. 2022 Aug 12;12(8):1070. doi: 10.3390/brainsci12081070 (PMC9406162; doi:10.3390/brainsci12081070)
Supplement: Supplementary file 1 [file brainsci-12-01070-s001.zip › brainsci-1820359-supplementary.pdf]

Supplementary Information

Supplementary Table S1. Results of linear regression models using 8-ISO and 8-ISO/LPH as predictor variables for termination sleep factors.

**Outcomes**	**Overall Model**				**Association with lipid peroxidation markers**			
	Adjusted R^2	Mean Square	F	P-value	Standardized Beta	Standard error	t	P-value
**Model 1 – unadjusted**								
**8-ISO**								
Sleep Efficiency	0.313	16.787	22.688	<0.001*	0.075	0.004	0.834	0.406
Perceived Sleep Quality	0.500	32.453	47.940	<0.001*	0.030	0.011	0.386	0.700
Daily Disturbances	0.023	68115.148	1.709	0.117	0.060	0.939	0.566	0.573
**8-ISO/LPH**								
Sleep Efficiency	0.261	13.110	16.926	<0.001*	0.067	0.012	0.437	0.467
Perceived Sleep Quality	0.452	24.966	37.774	<0.001*	0.118	0.011	1.474	0.144
Daily Disturbances	0.029	101893.819	1.888	0.098	-0.025	2.864	-0.242	0.810
**Model 2 – adjusted with demographics**								
**8-ISO**								
Sleep Efficiency	0.357	8.009	11.550	<0.001*	0.050	0.004	0.572	0.569
Perceived Sleep Quality	0.505	13.507	20.152	<0.001*	0.017	0.004	0.220	0.826
Daily Disturbances	0.015	53734.271	1.303	0.270	0.037	0.954	0.346	0.730
**8-ISO/LPH**								
Sleep Efficiency	0.311	6.591	9.120	<0.001*	0.016	0.012	0.178	0.859
Perceived Sleep Quality	0.459	10.509	16.084	<0.001*	0.092	0.012	1.110	0.370

Daily Disturbances	0.063	1.612	68767.605	0.165	-0.054	2.973	-0.497	0.620
**<u>Model 3 – adjusted with demographics and neuropsychiatric symptoms</u>**								
**8-ISO**								
Sleep Efficiency	0.174	3.892	3.892	<0.001*	0.003	0.005	0.720	0.474
Perceived Sleep Quality	0.212	5.645	4.662	<0.001*	0.022	0.005	0.220	0.826
Daily Disturbances	0.429	67969.50	1.723	0.113	0.053	0.933	0.509	0.612
**8-ISO/LPH**								
Sleep Efficiency	0.229	4.468	1.827	<0.001*	0.071	0.014	0.711	0.479
Perceived Sleep Quality	0.343	7.140	7.708	<0.001*	0.154	0.014	1.659	0.101
Daily Disturbances	0.528	75029.58	1.827	0.092	0.048	2.994	0.445	0.657

Demographic covariates: Age, Sex, BMI; Neuropsychiatric Symptom covariates: Hospital Anxiety and Depression scores
